# Statistical support for the hypothesis of developmental constraint in marsupial skull evolution

**DOI:** 10.1186/1741-7007-11-52

**Published:** 2013-04-26

**Authors:** C Verity Bennett, Anjali Goswami

**Affiliations:** 1 Department of Genetics, Evolution and Environment, University College London, London, UK; 2 Department of Earth Sciences, University College London, London, UK

**Keywords:** Marsupial, Placental, Cranium, Developmental constraint, Geometric morphometrics

## Abstract

**Background:**

In contrast to placental neonates, in which all cranial bones are ossified, marsupial young have only the bones of the oral region and the exoccipital ossified at birth, in order to facilitate suckling at an early stage of development. In this study, we investigated whether this heterochronic shift in the timing of cranial ossification constrains cranial disparity in marsupials relative to placentals.

**Methods:**

We collected three-dimensional (3D) landmark data about the crania of a wide range of extant placentals and marsupials, and from six fossil metatherians (the clade including extant marsupials and their stem relatives), using a laser scanner and a 3D digitizer. Principal components analysis and delta variance tests were used to investigate the distribution and disparity of cranial morphology between different landmark sets (optimizing either number of landmarks or number of taxa) of the whole skull and of individual developmental or functional regions (neurocranium, viscerocranium, oral region) for extant placentals and marsupials. Marsupial and placental data was also compared based on shared ecological aspects including diet, habitat, and time of peak activity.

**Results:**

We found that the extant marsupial taxa investigated here occupy a much smaller area of morphospace than the placental taxa, with a significantly (*P*<0.01) smaller overall variance. Inclusion of fossil taxa did not significantly increase the variance of metatherian cranial shape. Fossil forms generally plotted close to or within the realm of their extant marsupial relatives. When the disparities of cranial regions were investigated separately, significant differences between placentals and marsupials were seen for the viscerocranial and oral regions, but not for the neurocranial region.

**Conclusion:**

These results support the hypothesis of developmental constraint limiting the evolution of the marsupial skull, and further suggest that the marsupial viscerocranium as a whole, rather than just the early-ossifying oral region, is developmentally constrained.

## Background

Fossil and molecular estimates generally agree that the lineages leading to marsupial and placental mammals diverged over 160 million years ago (Ma), in the Late Jurassic period [1,2]. Despite this shared time of origin as sister clades, recent marsupials and placentals differ markedly in taxonomic diversity and geographical range. Whereas placentals number over 5,000 species and are globally distributed, extant marsupials are far less speciose, with 331 species, and occupy only Australasia, South America, and Central America, with one species in North America [3], yet the marsupial (and broader metatherian) fossil record demonstrates that this now depauperate and geographically restricted clade previously had a global distribution [4-7]. The differences in the evolutionary histories of these two clades, and how these differences have contributed to their disparate modern diversities, has been a topic of research and debate for decades, but there is as yet little consensus on the relative importance of intrinsic factors such as development, and extrinsic drivers such as competition or geography [8-10]. In this study, we investigated the evidence for the role of developmental constraints on marsupial evolution.

The most obvious difference between marsupial and placental mammals is, as their clade names suggest, their developmental mode. Marsupials are born in a highly altricial state, and must immediately travel to their mother’s teat, which is often located in the pouch [11]. Once they attach to a nipple, marsupial young undergo a much longer period of development. The nature of the obligatory, independent journey to the pouch varies across marsupial clades. For didelphimorphians (opossums) and some diprotodontians (including possums and kangaroos), which have forward-facing pouches, an upwards, forelimb-powered crawl is required. In peramelimorphians (bandicoots) and some diprotodontians (such as wombats), which have backwards-facing pouches, and in dasyuromorphians (marsupial carnivores and mice), which have open pouches, the journey to the pouch is a downwards, sinusoidal slither, aided by the positioning of the mother (Wilson and Reeder [3] and references therein). These mechanical demands do not end with arrival in the pouch, as once the neonate is attached to the teat, it must satisfy the mechanical demands of suckling to survive. To fulfill this function, marsupial skulls at birth are necessarily ossified in the oral region (including the anterior portion of the mandible, premaxillae, maxillae, palatines, and pterygoids) for feeding, and the exoccipital region for movement of the head relative to the spine, whereas the remaining cranial bones ossify after birth [12]. By contrast, even the most altricial neonates of their placental sister group are born at a much later stage of development, with all or nearly all cranial bones at least partially ossified prior to the commencement of suckling.

It has long been hypothesized and debated that early functional demands have constrained the evolution of novel morphologies in marsupial phenotypes [9,13,14] with particular regard to the lack of fully volant or aquatic marsupial species. In a study quantifying ontogenetic changes in the shoulder girdle and comparing adult diversity in the scapula and pelvis, Sears [15] found evidence for constraint in marsupial shoulder-girdle morphology produced by this early functional requirement. There is also evidence that the early crawl constrains forelimb morphology in marsupials [16,17].

Whether or not the morphology of the marsupial skull is also constrained by these early functional demands requires the comparison of adult morphology, the end product of development. A few previous studies have quantitatively compared disparity in adult cranial morphologiy across placentals and marsupials, but all of these have focused almost exclusively on carnivorous taxa [18-21]. Goswami *et al*. [21] found no evidence for cranial constraint when comparing the adult morphological variance of extant and extinct metatherian and eutherian hypercarnivores, although the early-ossifying oral apparatus was not assessed separately. By contrast, Prevosti *et al*. [22] found that disparity in mandible morphology is more constrained in extant carnivorous marsupials than in the Carnivora; however, the exclusion of extinct forms from that analysis left the most specialized marsupial carnivores unsampled. Marsupial ecology extends far beyond carnivory. For example, members of Diprotodontia, the most taxonomically diverse marsupial order today, are mostly folivores (including browsers and grazers), although there are also many frugivores, insectivores, and omnivores, and, in the recent past, carnivores within this clade. Whether the skull or mandible shows evidence for developmental constraint in marsupials representing ecological groups other than carnivores has yet to be tested.

In this study, we quantitatively tested the hypothesis of developmental constraint in the marsupial cranium across marsupial phylogeny and across diverse ecologies (diet, habitat, and time of activity) using geometric morphometrics. It is important to consider all of these aspects and to sample the full range of marsupial ecology in order to make meaningful comparisons between marsupial and placental diversity. Specifically, we tested whether marsupials show significantly less cranial disparity than placentals across the entire skull and within relevant developmental and functional sub-regions (viscerocranium, neurocranium, oral apparatus). The independent comparison of cranial sub-regions allows assessment of whether any observed differences in disparity between marsupials and placentals are driven specifically by the early-ossifying regions of the skull (that is, the oral apparatus). We further test whether the addition of well-preserved fossil metatherians to the dataset would significantly increase the disparity measured from extant marsupials alone.

## Results

### Principal components analysis

The following results describe the first four principal components (PCs) of each analysis, as subsequent PCs did not explain a sufficiently large percentage of the variation to warrant meaningful discussion (see Additional file 1: Table S1).

**Figure 1:**
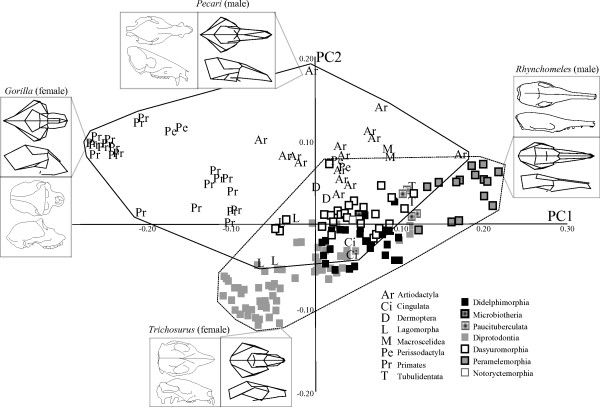
**Principal component (PC)s 1 and PC2 and selected wireframes and line drawings for the ‘maximum landmarks’ dataset.** (Top) dorsal and (bottom) lateral views. Letters represent placental taxa, as described in the key; squares represent marsupials. Solid line indicates the range of morphospace occupied by placental taxa; dashed line represents occupied morphospace for marsupials.

**Figure 2:**
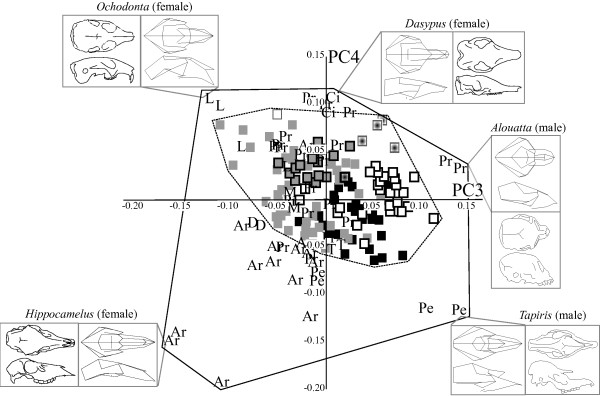
**Principal components (PC)3 and 4 and selected wireframes and line drawings for the ‘maximum landmarks’ dataset.** (Top) dorsal and (bottom) lateral views. Symbols as in Figure 2.

In the extant-only ‘maximum landmarks’ dataset (Figure 1) PC1 (35% of the variance) separated the long-snouted, narrow skulls of peramelemorphians from the flat-faced, wider and taller skulls of primates. PC2 in this dataset (12% of the variance) separated the longer, dorsoventrally shorter skulls of artiodactyls from the taller, anterioposteriorly shorter skulls of diprotodontians. Using these first two PCs, many diprotodontians and a few didelphimorphians fell outside of placental space, but all dasyuromorphians fell within the range of placental morphospace. PC3 and PC4, each accounted for 8% of the total variance (Figure 2), showed far less phylogenetic clustering and greater overlap between orders. On these axes, marsupial morphospace was entirely within placental space.

**Figure 3:**
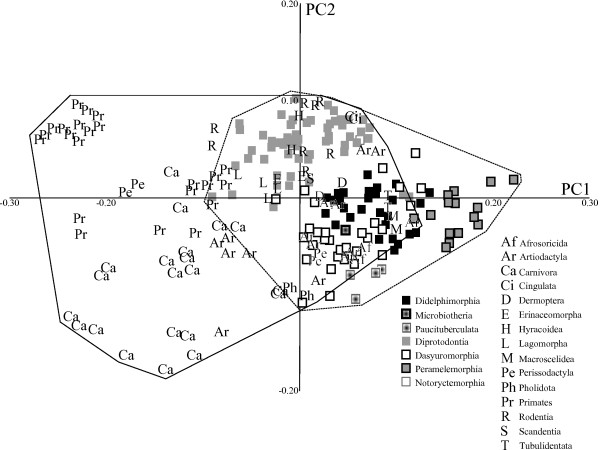
**Principal components (PC)1 and 2 for the ‘maximum taxa’ dataset.** Symbols as in Figure 2.

**Figure 4:**
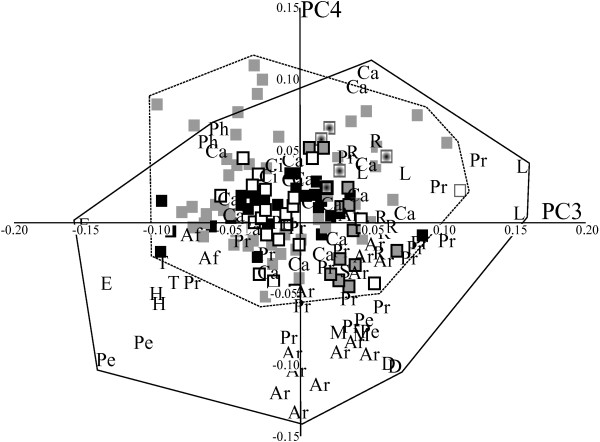
**Principal components (PC)3 and 4 for the ‘maximum taxa’ dataset.** Symbols as in Figure 2.

In the ‘maximum taxa’ dataset (Figure 3), PC1 accounted for 39% of the variance, and showed the same separation between long-snouted and flat-faced skulls as in the ‘maximum landmarks’ dataset. PC2 (14% of the variance) separated the long-snouted, narrower skulls of the pangolin from the wider, taller skulls of diprotodontians in the ‘maximum taxa dataset’. PC3 and PC4 (10% and 7% of the total variance, respectively; Figure 4) also showed more overlap between marsupial and placental morphospace than PC1 and PC2, and only a few diprotodontians fell outside of placental space.

Taxa largely clustered by phylogenetic relationship in the morphospace described by these first two PCs for both extant-only datasets. Shape changes on axes 3 and 4 were far subtler than for PC1 and PC2. There was extensive overlap between the marsupial and placental morphospaces in both datasets, and placentals occupied a larger area of morphospace in all PC analysis plots.

The placental orders Afrosoricida, Tubulidentata, Pholidota, Lagomorpha, Hyracoidea, Cingulata, Dermoptera, Scandentia, Rodentia, Erinaceomorpha, and Macroscelidea all fell within or very close to the region of the morphospace occupied by marsupials. Some artiodactyls and perissodactyls also fell near marsupials in the major axes of the morphospace. Although there was some overlap, Primates and Carnivora fell furthest away from the marsupials in both analyses.

When the ‘maximum taxa’ dataset was subdivided into ecological groupings, marsupials again inhabited a relatively smaller region of morphospace on PC1 to PC4, and overlapped entirely with placentals (see Additional file 2: Figure S2; see Additional file 3: Figure S3; see Additional file 4: Figure S4) for nearly all the ecological groups. The sole exception to this pattern was found in the analysis of fossorial taxa, in which placentals and marsupials occupied roughly equal areas of morphospace.

### Delta variance tests

Both the ‘maximum taxa’ and ‘maximum landmarks’ datasets showed significantly greater (*P*<0.01) morphological disparity (higher variance) in placentals than in marsupials when the full skull was considered (Table 1). In the comparison of developmentally and functionally significant cranial sub-regions, the viscerocranium including the oral region, the viscerocranium excluding the oral region, and the oral region alone all showed significantly (*P*<0.01) greater morphological disparity in placentals than marsupials, and these differences remained after Bonferroni correction. However, there was no significant difference between marsupials and placentals in disparity of the neurocranial region.

**Table 1:** Delta variance test results for extant taxa datasets for extant marsupials versus placentals

**Dataset**	**Skull region**	**Marsupial variance**	**Placental variance**	**Delta variance**	***P*-value**^**a**^
Maximum landmarks	Whole skull	0.0258	0.0681	0.0423	<<0.001
	Neurocranium	0.0344	0.0422	0.0078	0.329
	Viscerocranium	0.0308	0.0873	0.0565	<<0.001
	Non-oral viscerocranium	0.0170	0.0446	0.0275	<<0.001
	Oral region	0.0174	0.0572	0.0398	<<0.001
Maximum taxa	Whole skull	0.0178	0.0521	0.0344	<<0.001
	Neurocranium	0.0342	0.0504	0.0162	0.150
	Viscerocranium	0.0180	0.0697	0.0516	<<0.001
	Oral region	0.0044	0.0251	0.0207	<<0.001
Folivores	Whole skull	0.0004	0.0015	0.0011	0.027^b^
	Neurocranium	0.0204	0.0409	0.0205	0.048^b^
	Viscerocranium	0.0122	0.0649	0.0528	<<0.001
	Oral region	0.0019	0.0198	0.0179	<<0.001
Frugivores	Whole skull	0.0094	0.0558	0.0464	0.001
	Neurocranium	0.0235	0.0433	0.0198	0.269
	Viscerocranium	0.0110	0.0851	0.0740	0.006
	Oral region	0.0019	0.0130	0.0112	0.006
Omnivores	Whole skull	0.0130	0.0439	0.0309	<<0.001
	Neurocranium	0.0314	0.0310	-0.0004	0.966
	Viscerocranium	0.0092	0.0580	0.0488	<<0.001
	Oral region	0.0037	0.0347	0.0310	<<0.001
Carnivores/insectivores	Whole skull	0.0186	0.0535	0.0348	0.034^b^
	Neurocranium	0.0417	0.0809	0.0392	0.280
	Viscerocranium	0.0165	0.0719	0.0554	0.001
	Oral region	0.0031	0.0225	0.0193	<<0.001
Nocturnal/crepuscular	Whole skull	0.0181	0.0360	0.0179	<<0.001
	Neurocranium	0.0386	0.0296	-0.0090	0.413
	Viscerocranium	0.0186	0.0438	0.0251	<<0.001
	Oral region	0.0044	0.0198	0.0154	<<0.001
Arboreal	Whole skull	0.0107	0.0347	0.0240	<<0.001
	Neurocranium	0.0156	0.0280	0.0123	0.031^b^
	Viscerocranium	0.0135	0.0392	0.0257	<<0.001
	Oral region	0.0028	0.0139	0.0111	<<0.001
Terrestrial	Whole skull	0.0175	0.0484	0.0309	<<0.001
	Neurocranium	0.0347	0.0628	0.0281	0.097
	Viscerocranium	0.0182	0.0660	0.0478	<<0.001
	Oral region	0.0060	0.0164	0.0103	0.001
Fossorial	Whole skull	0.0198	0.0314	0.0116	0.103
	Neurocranium	0.0380	0.0296	-0.0089	0.454
	Viscerocranium	0.0180	0.0275	0.0095	0.203
	Oral region	0.0040	0.0143	0.0103	0.033^b^

^a^*P*-values are before Bonferroni correction.

^b^No longer significant after Bonferroni correction.

Similar results were obtained when taxa were divided into ecological groups, with the exception of marginally significant differences in neurocranial disparity between arboreal (*P* = 0.03) and folivorous (*P* = 0.048) marsupials and placentals; however, these exceptions were not supported after Bonferroni correction. There were marginally significant differences between placentals and marsupials in the disparity of the entire skull for folivorous (*P* = 0.027) and carnivorous (*P* = 0.034) forms, but again, not after Bonferroni correction, whereas all other ecological groups showed significantly different (*P*<0.01) disparity between marsupials and placentals. All three groups of viscerocranial landmarks showed significantly higher disparity in placentals than in marsupials when ecological groups were compared separately, with the exception of fossorial forms. Fossorial marsupials and placentals showed no significant difference in viscerocranial disparity, except for a marginally significant difference (before Bonferroni correction) in the oral region (*P* = 0.033). After Bonferroni correction, no set of viscerocranial landmarks showed significant differences between fossorial marsupials and placentals.

### Fossil taxa

**Figure 5:**
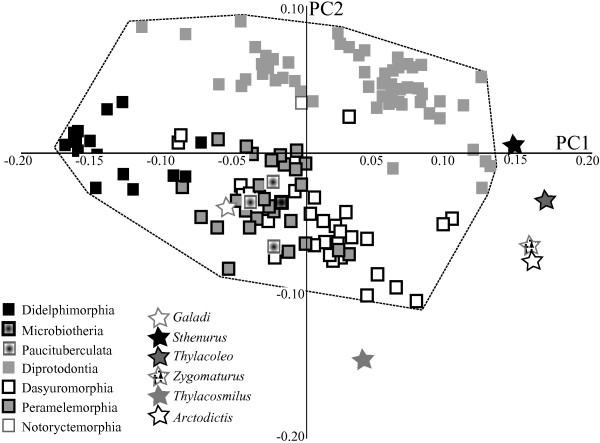
**Principal components (PC)1 and PC2 for the extinct and extant metatherians dataset.** Squares represent extant taxa, stars represent fossil taxa, and dashed line indicates the range of morphospace occupied by extant metatherians.

When fossil marsupials are added into the analysis, five of the six fossil taxa were found to fall outside the region of morphospace of PC1 (37% variance) and PC2 (18% variance) occupied by Recent marsupials (Figure 5). *Galadi*, the Oligo-Miocene bandicoot, falls with other peramelemorphians, whereas *Sthenurus* falls close to other diprotodontians. The remaining fossil diprotodontians, *Thylacoleo* and *Zygomaturus*, plotted more distantly to other diprotodontians, and the sparassodont *Arctodictis* plots very closely to *Zygomaturus*, and much farther from the only other sparassodont included in this study, the sabre-toothed marsupial *Thylacosmilus atrox*. Inclusion of these fossil taxa with recent forms did not significantly increase the morphological variance of the metatherian dataset (Table 2) or change the results of the delta variance permutation tests comparing marsupial (and non-marsupial metatherian) and placental disparity.

**Table 2:** Delta variance test results for extant marsupials versus extant marsupials plus fossil metatherian taxa

**Skull region**	**Marsupials variance**	**Marsupials and fossils variance**	**Delta variance**	***P*-value**
Whole skull	0.0165	0.0187	0.0022	0.205

## Discussion

Despite the exclusion of several placental taxa with unusual cranial morphologies (notably whales, bats, and elephants), and the limited fossil taxa included, we found in the current study that marsupial crania are, on the whole, significantly less disparate compared with placentals. Although inclusion of the enigmatic *Tarsipes* could potentially increase the variance of the extant marsupials in future studies, we are confident that this one taxon would not alter the substantial difference in variance between marsupials and placentals reported here. Thus, the results of this study support the hypothesis that marsupial crania are developmentally constrained, and that this constraint is likely to have limited the morphological evolution of marsupials relative to their placental sister groups. In particular, the observation that the viscerocranial region, which includes the early-ossifying bones of the oral region, is significantly less disparate in marsupials than in placentals, whereas the late-ossifying neurocranial region has similar disparity in both clades, is consistent with the hypothesis that the differential evolutionary success of these two groups was shaped by developmental strategy rather than by extrinsic factors.

### Developmental timing, integration, and lability

Although relative cranial ossification sequence is largely conserved across mammals [23,24], there is a delay in raw timing between the development of bones in the oral region and those in the neurocranium of marsupials compared with placentals [25-27]. Anterior elements of the skull also tend to show less heterochronic variation and basicranial elements show the most [23]. As we tested here, these differences in raw timing and rank variability in ossification sequences correlate with differential cranial disparity for the viscerocranial and neurocranial regions, with the former showing significantly less disparity in marsupials than in placentals. These regional differences in amount of heterochronic variation may possibly relate to the different evolutionary lability of these regions [23], although this hypothesis has yet to be tested with quantitative data on ontogenetic or morphological variation.

Post-weaning ontogeny of marsupial cranial morphology has been studied in several omnivorous and carnivorous species [28-32]. These studies have shown the existence of some common developmental patterns across marsupials, including a faster-growing viscerocranium than neurocranium in early post-weaning development, negative allometry across the entire braincase and in the height of the occipital plate, and positive allometry in the height of the dentary [31]. That these major aspects of post-weaning growth differentiate skull regions is suggestive of the modular nature of cranial development [33-35]. Moreover, that a common growth pattern was found across the full viscerocranium, rather than only in the early-ossifying oral bones of this region, suggests that this region is developmentally integrated, and provides a possible mechanism by which the functional constraints imposed on the oral bones translate to the lower disparity across the entire viscerocranium seen in the current study.

A recent study [35] found that cranial variance in a marsupial (*Monodelphis*) remained constant through ontogeny, whereas in a placental (*Cryptoprocta*), variance decreased markedly from the early to the later stages. Moreover, *Monodelphis* also showed a decrease in integration of the oral region through ontogeny. This led to a tentative hypothesis by the authors that the combination of high integration of the oral region early in ontogeny, alongside functional demands on those early-developing oral bones, may result in low and constant variance of that region through marsupial ontogeny, in contrast to placentals. Although further data are needed to test that hypothesis, a possible extension suggested by the data from the current study could be that, if the marsupial skull is indeed constrained in the oral region during early development, and if the viscerocranial elements of the skull are strongly integrated, as some studies have suggested [36-38], then the remainder of the viscerocranium (for example, those elements that do not ossify early in development) would also be likely to be constrained as a result of integration, rather than by direct developmental or functional constraint.

Not only is the developmental strategy of marsupials very different to that of placentals, but the nature and timing of development also varies between marsupial groups. Peremelemorphs in particular represent the most unusual condition among extant marsupials in having evolved a chorioallantoic placenta, convergent with that of placentals, and also showing the fastest developmental rate of all marsupials [36]. Peramelemorphians also lack a pronounced crawl, as noted above, and this divergent strategy is reflected in their scapular ontogeny, which has been shown to differ significantly from that of other marsupials [15]. Interestingly, the PC analyses presented here show that peramelemorphians fall further outside of placental cranial morphospace than do other marsupial clades. For this reason, we hypothesize that the unusual development of peramelemorphians (which have a much shorter period during which the oral region develops and functions in isolation from the rest of the quickly developing viscerocranium and the rest of the skull), is related to the evolution of their distinct cranial morphology, relative to that of other marsupials. Future work on peremelemorphian cranial ontogeny thus represents an interesting avenue for research to further address the role of ossification timing and functional constraints on cranial evolution. A specific question of interest is whether peramelemorphians follow or deviate from the developmental trajectory of *Monodelphis*, discussed above, which is often used to represent a generalized marsupial condition.

It is important to remember that we cannot say for certain whether extinct forms shared the unique developmental strategy of extant marsupials, or indeed, with which group of marsupials they shared most developmental similarities. Of particular interest in this regard are the sparassodonts, included in this analysis and previous studies [21]. These form a group of South American metatherians of uncertain phylogenetic position, although recent analyses have placed them outside of crown marsupials [37]. It is possible that these taxa do not share the same developmental strategy, and thus might not be subject to the same developmental constraints on morphology, as crown marsupials. Nonetheless, the inclusion of fossil taxa did not significantly affect our results, and so we tentatively suggest that the developmental constraint hypothesis may apply to all of the metatherian clades sampled here.

### Comparisons with previous studies

Our findings are consistent with those of Prevosti *et al*. [22] who also found evidence to support the hypothesis of morphological constraint in the oral region (albeit in the dentary, not tested here) of extant marsupial carnivores. Functionally, this is logical, as the upper and lower jaws should be morphologically coupled for both mechanical and developmental reasons. Conversely, the results of this study concerning the marsupial carnivore/insectivore skull are in contrast to those of Goswami *et al*. [21], who included a broader sampling of living and extinct insectivorous and carnivorous species. Because our study compared only ecological groupings of recent taxa, it is possible that this disagreement indicates a greater diversity of carnivorous/insectivorous metatherians in the fossil record than in the present. Indeed, many carnivorous marsupials (or, more generally, metatherians) have become extinct relatively recently [38]. The inclusion of six metatherian fossil taxa (three of which are probably carnivorous/insectivorous) with the Recent marsupials in this study similarly did not significantly increase the overall variance. However, ecological groups were not analyzed separately when fossils were included, nor were placentals and metatherians, as no fossil eutherians (the clade including placentals and their stem relatives) were sampled. Thus, the difference in results between these studies may be a reflection of the limited sample sizes of the fossil taxa and the much broader sampling of extant taxa and different ecological groups in the analysis presented here. Nonetheless, developmental constraints may limit variation, but need not represent absolute barriers to evolution. Even if metatherian and placental disparity is comparable in a few ecological groups (possibly carnivorous and fossorial taxa), the results presented here suggest that a developmental constraint has limited marsupial cranial evolution for most, if not all, of the history of this clade.

Expanding these studies to include fossils representing other ecological groups is central to assessing whether fossil metatherians were subject to similar constraints to those found here for extant forms. For example, it has been shown that large placental omnivores, but not hypercarnivores, have been constrained, in terms of taxonomic diversity, on the southern continents since the late Oligocene [39]. Whether such a pattern also applies to metatherians, possibly in combination with the geographic constraint hypothesis discussed further below, is a promising avenue for future study that will benefit from a broadening of focus beyond comparisons of carnivorous fossil metatherians.

### The metatherian fossil record and alternative hypotheses for differential mammalian diversity

It has been suggested that functional requirements around birth bear little relevance to adult metatherian morphology [13,40], and that other factors are primarily responsible for the observed differences in marsupial and placental diversity. The first alternative hypothesis concerns the relative ages of crown placentals and crown marsupials, while an alternative hypothesis is that the diversification of metatherians has been limited by their biogeographical history and resulting ‘isolation’ on the southern continents.

The earliest metatherians (marsupials and their closest fossil relatives) are known to have existed from the Early Cretaceous of China [4], and may have been restricted to the northern continents, with an especially rich record in North America, until the end Cretaceous, although there are some debated occurrences in Africa and Madagascar [41,42]. Non-marsupial metatherians (or possible early didelphimorphs), such as herpetotheriids continued to inhabit the northern continents, although at much reduced numbers, well into the Cenozoic [7,43]. Molecular approaches estimate the first divergences of the extant marsupial clades around 69 Ma, with the divergences of the Australian orders occurring around 60 Ma [44]. The first paleontological evidence for the extant orders is found in the Paleocene (around 65 to 63.3 Ma) of North America with the appearance of the peradectids, the first known members of Didelphimorphia. Didelphimorphia, Paucituberculata, and Microbiotheria appear in the Palaeocene (around 64.5 to 62.5 Ma) of Brazil. Marsupials first appear in scarce numbers in the fossil record of Australia in the early Eocene [45], but it is not until the prolific Riversleigh deposits of the Oligo-Miocene that all remaining extant marsupial orders (Diprotodontia, Notoryctemorphia, Peramelemorphia, and Dasyuromorphia) appear. However, a more precise understanding of Gondwanan metatherian biogeography, particularly with regard to the biogeographic origin of the enigmatic Microbiotheria (a small clade of South American marsupials that is a sister group to Australodelphia) is yet hindered [46] in large part by an extremely poor pre-Oligocene terrestrial vertebrate record from Australia and Antarctica.

Placental phylogenetics and biogeography are somewhat better understood, with a growing body of evidence over the past decade supporting the division of modern placental clades into four superorders, Afrotheria, Xenarthra, Laurasiatheria, and Euarchontoglires, with the latter two combined in Boreoeutheria [47-53]. This divergence of superorders is thought to have been near-simultaneous, and has been linked, albeit contentiously, to their semi-isolation in Africa, South America, and Laurasia, respectively [52] (although this hypothesis is not congruent with the presence of early afrotherians in the fossil record of North America [54]). According to recent molecular divergence date estimates, the placental superorders diverged around 88 to 90 Ma, but most extant orders seem to have originated near to or soon after the Cretaceous-Paleogene extinction, around 65 Ma [2,55]. Although there is as yet no confirmed paleontological evidence for crown placentals in the Late Cretaceous (Nishihara *et al*. [51] Meredith *et al*. [52], and references therein), crown placentals are known from the earliest Paleocene (around 63 to 64 Ma) of North America, and are found on most continents by the mid Paleocene.

The difference in timing between the basal divergences of crown marsupials and crown placentals has been suggested as one reason for the lower diversity seen in marsupials [40], although the difference in crown-clade age is out of proportion with the difference in taxonomic diversity between these clades. It has also been suggested that marsupials were hit harder by the K-Pg mass extinction than were placentals (or their respective stem groups), but this has never been explicitly tested. Moreover, paleontological evidence suggests that both groups experienced great losses in diversity, with most Cretaceous metatherian and eutherian families becoming extinct during that event [56-59].

The second alternative, the geographic constraint hypothesis, is based on the observation that placentals are currently more taxonomically diverse throughout the northern continents, and that the northern continents have been in more frequent contact during the Cenozoic [8,10]. Indeed there are many episodes of dispersal among North America, Asia, and Europe, but both Australia and, until the closure of the Isthmus of Panama (around 3 Ma [59], but see Montes *et al*. [60]), South America, have been almost entirely isolated since the final breakup of Gondwana and opening of the Drake Passage, around 30 Ma [61]. If competition and faunal exchange drive evolution, then geographic isolation and lack of competition may certainly contribute to the current state of marsupial taxonomic diversity. The relatively low diversities of the ‘southern’ placental superorders Xenarthra and Afrotheria may provide further evidence for the possible, but as of yet untested, importance of geographic isolation.

More importantly, however, neither clade age nor the geographic constraint hypothesis can account for the differential disparities of the viscerocranium and neurocranium described here. If extrinsic factors are primarily responsible for the low taxonomic diversity and low morphological disparity of marsupials, then all regions of the skull, not just the early-ossifying viscerocranial elements, should show lower disparity in marsupials than in placentals. The results of the study presented here are consistent with the hypothesis of developmental constraint in the marsupial skull, but do not exclude the possibility of some geographical component also limiting metatherian evolution. Ideally, future work combining both aspects would more fully sample from the metatherian fossil record, including that of the northern continents, but at present, there is a paucity of complete and undeformed metatherian cranial material from those regions.

## Conclusions

More fossil data representing the full range of metatherian ecology, as well as quantitative developmental data, are necessary to further test both the hypothesis of cranial constraint in marsupials and the alternative hypothesis. However, the results of this study are consistent with the hypothesis that a developmental constraint imposed by the marsupial reproductive strategy of short gestation and long lactation periods has limited the cranial disparity in this clade of mammals. In particular, the observation that marsupials are less disparate than placentals in viscerocranial morphology, but are equally disparate in neurocranial morphology, is highly suggestive that the early ossification and use of the oral apparatus in marsupials is the specific driver of the differential disparities of these clades. Lastly, our preliminary data for fossil metatherians suggests that this constraint may also have applied to the broader clade and is not limited to crown group marsupials; however, this is a limited sample and should be interpreted with caution.

Future work should endeavor to expand fossil sampling by exploring methods that are not reliant on identifying comparable landmarks across a wide range of taxa, because poor preservation limits the availability of complete specimens and can obscure sutures. Different methods of morphological data capture and analysis are currently being explored in order to enable the inclusion of damaged or partial fossil skulls. These improvements will enable a more robust investigation of this constraint in extinct marsupials and their stem relatives, and will further elucidate the patterns and process that have shaped the evolution of metatherian diversity.

## Methods

### Specimens

**Figure 6:**
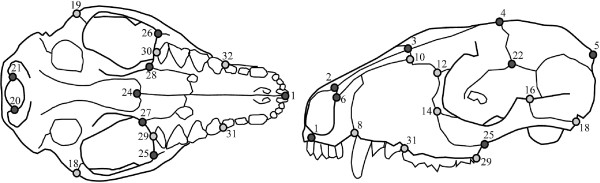
**Location of cranial landmarksviews used in the ‘maximum taxa’ (light grey only) and ‘maximum landmarks’ (light and dark grey) datasets.** (Left) ventral and (right) lateral. Numbers correspond to Table 1.

Landmark data (Figure 6, Table 3) were collected using a digitizer (Immersion MicroScribe G2X; Immersion Corp., San Jose, CA, USA) and a laser scanner (NextEngine; NextEngine Inc., Santa Monica, CA, USA). Skulls from 125 species of therian mammals (see Additional file 5: Table S5) were used in this study, including where possible a male and a female with the same provenance for each selected species. Species were selected using a random-number generator to choose one species from every marsupial genus and one genus from every placental family, in accordance with Wilson and Reeder’s mammalian species list [3]. If that species was not available in international museum collections, the next one on the list was selected. Some taxa were excluded from the study because of lack of availability of a complete undamaged adult skull (for example, *Tarsipes*, the honey-possum), lack of enough clearly homologous landmarks (for example, animals with heavily fused skulls such as bats and some carnivorans, or animals with widely divergent cranial morphology, such as whales), or inability to landmark the skull in just two views. Elephant and rhino skulls were excluded because of their large size, as stitching several ‘patches’ of overlapping landmarks to fully cover the cranium would have increased the error relative to all other skulls. Although these exclusions primarily involved unusual placentals and thus may have reduced estimates of placental cranial disparity, our sampling did include the vast majority of extant placental diversity, particularly in the terrestrial realm cohabited by marsupials.

**Table 3:** **Cranial landmark descriptions and assignment to skull regions for disparity analyses**^a^

**Landmark number**^b^	**Landmark description**	**Skull region**
1^c,d^	Midline point between the premaxillae and the upper central incisors	Oral
2^c,d^	Midline anterior-most point of the nasal-nasal suture	Viscerocranium (non-oral)
3^d^	Midline posterior point of the nasals in contact with frontals	Viscerocranium (non-oral)
4^d^	Midline posterior point of the frontals in contact with parietals	Neurocranium
5^c,d^	Midpoint at posterior-most extent of vault	Neurocranium
6 and 7^c,d^	Anterior nasal-premaxilla/maxilla (nasal opening), left and right	Viscerocranium (both oral and non-oral)
8 and 9^c^	Premaxilla-maxilla suture on the alveolar lateral margin, left and right	Oral
10 and 11	Nasal-frontal-maxilla/premaxilla suture junction, left and right	Viscerocranium (both oral and non-oral)
12 and 13	Medial-most maxilla-lacrimal contact, left and right	Viscerocranium (both oral and non-oral)
14 and 15	Lateral most maxilla-lacrimal contact, left and right	Viscerocranium (both oral and non-oral)
16 and 17^c^	Jugal-squamosal (dorsal zygomatic arch), left and right	Viscerocranium (non-oral)
18 and 19^c^	Jugal-squamosal (ventral zygomatic arch), left and right	Viscerocranium (non-oral)
20 and 21^c,d^	Dorsal most occipital condyle-foramen magnum margin, left and right	Neurocranium
22 and 23^c^c	Ventral-most extent of frontal-parietal suture, left and right	Neurocranium
24^c^	Midline posterior point of the palatine-palatine suture	Oral
25 and 26^c,d^	Ventral-most point of the jugal-maxilla suture	Viscerocranium (non-oral)
27 and 28^d^	Posterior-most maxilla-palatine junction on ventral surface, left and right	Oral
29 and 30^c^	Posterior lateral extent of molar row	Oral
31 and 32^c^	Anterior lateral extent of molar row	Oral

^a^Fossil skulls investigated here included the Oligo-Miocene peramelmorphian *Galadi speciosus*, the Quaternary diprotodontians *Sthenurus occidentalis*, *Zygomaturus trilobus*, and *Thylacoleo carnifex*, and the sparassodonts *Thylacosmilus atrox* (Miocene) and *Arctodictis sinclari* (Eocene).

^b^Numbers correspond to Figure 1.

^c^Landmarks used in ‘maximum fossil’ dataset

^d^Landmarks used ‘maximum taxa’ dataset.

### Landmarks

Landmarks were selected to represent clearly homologous points, such as suture junctions or extreme points of curvature, and to fully sample the morphology of the entire skull (Figure 6, Table 3). The number of landmarks used was largely limited by the extent of fusion of the skull bones to one another, limiting the ability to identify sutures. This fusion was mostly a problem in the neurocranium and basicranium, and mainly affected placental musteloid carnivorans and some fossorial forms. Sutures also vary across mammal groups (and sometimes within species or even specimens) in terms of which bones are in contact. The most variable of these sutures were necessarily excluded from this study. Others were included in a subset of the data (see below). In some cases, it was possible to relax the description of the landmark to make it applicable across a larger range of taxa; for example, ‘the ventral extent of the frontal-parietal suture’ does not make it necessary to specify whether this point on the skull is contacting the alisphenoid or squamosal bone.

Three configurations of landmark data were analyzed. A ‘maximum landmark’ dataset maximized homologous landmarks (n = 32) and included 8 (of the 20 extant) placental and all seven marsupial orders, and a total of 104 species. A ‘maximum taxa’ dataset used a reduced number of landmarks (n = 16) and included 15 (of the 20 extant) placental orders and all 7marsupial orders, giving a total of 125 therian species. The ‘maximum fossils’ dataset used a landmark set (n = 20) intermediate between that of the ‘maximum landmark’ and ‘maximum taxa’ datasets to optimize the number of fossil taxa that could be compared with Recent marsupial taxa. This last dataset included the six fossil taxa described above (see Additional file 5: Table S5), and 7 extant marsupial orders, to give a total of 82 living and extinct species. All fossils used in this study were considered accurate representations of the original skull shape free from deformation, as they did not exhibit considerable asymmetry. The inclusion of extinct metatherians investigated whether extant diversity is contradicted in the fossil record and is a particular condition of the present. Investigation of extinct eutherian diversity was beyond the scope of this study.

### Ecological groups

The extant ‘maximum taxa’ landmark data was further separated into eight ecological groups based on dominant diet type, time of activity and habitat, using information sourced from the Animal Diversity Web [62]: folivore, frugivore, carnivore/insectivore, omnivore, nocturnal/crepuscular, arboreal, fossorial, and terrestrial (see Additional file 5: Table S5). Insectivores and carnivores were combined into one dietary group because many insectivorous marsupial taxa also regularly consume small vertebrates and this dietary distinction is largely related to the size of the animal. Because only 11 taxa are known to be crepuscular, and 6 of these are also reported to be nocturnal, the crepuscular and nocturnal taxa were combined into one group. Because only two marsupial taxa (*Myrmecobius* and *Hypsiprymnodon*) are truly diurnal, and only two marsupial taxa are undisputedly cathemeral (*Aliurops* and *Dasycercus*), these categories were removed from further analysis. Despite exclusion of diurnal and cathemeral taxa, the nocturnal/crepuscular dataset still provided useful comparisons between marsupials and placentals based on time of most frequent activity.

### Data analysis

Prior to all analyses, Procrustes superimposition was used to remove the size and orientation components of the data, leaving only shape. Next, PC analysis was performed to examine distribution and overlap of the clades of interest in the cranial morphospace. Allometry was removed in an attempt to avoid biasing results by the smaller size range and average size of marsupial skulls compared with those of placentals. This was achieved by removing the component of variation explained by difference in centroid size, determined by regressing log centroid size against initial PC scores. The residual of this regression (that is, the components of variation not explained by size alone) were then subjected to a second PC analysis.

Before carrying out comparisons of disparity, sampling issues must be addressed. Variance-based disparity measures, such as those used here, are more robust to sample size than range-based metrics [63], but we further corrected for differences in sample size between the marsupial and placental datasets by bootstrapping male and female marsupials and placentals to the size of the smallest dataset before quantifying and comparing disparity. We conducted 1,000 iterations of the bootstrapping procedure in order to produce results robust to differences in sample size.

To compare cranial disparity between groups across the entire skull and within specific cranial regions, a delta variance test was used. This approach tested for significant differences in the variance of two groups, marsupials and placentals, compared with the variance expected if the taxa sampled were randomly assigned to a group, essentially deciding whether the difference in variance between the marsupial and placental groups is different to that which would be generated by any random grouping of the sampled taxa. To generate the null expectation, the residual Procrustes distances of individual taxa from the mean of each group (after Procrustes superimposition) were randomly permuted and reassigned to the two groups. This process was repeated 1,000 times, and the resulting differences in variance in the permuted datasets were compared with the original differences in variance observed between marsupials and placentals to determine if the observed differences were significantly greater than the random expectation. The following cranial regions were compared: entire cranium, neurocranium, oral region, viscerocranium, and viscerocranium excluding any elements of the oral region. All analyses were conducted using the statistical programming software ‘R’ [64] using the software packages shapes [65] and a bind [66], as well as some custom-written code (see supplementary information). Significance was set at *P*<0.01 for all analyses, with a Bonferroni correction of 5 and 36 for the maximum landmarks and maximum taxa (including ecological splits) datasets, respectively, to account for repeated use of the same data.

Phylogenetically corrected analyses were not performed, as the goal of the project was to compare the disparity of two monophyletic sister clades, hence the phylogenetic component of their morphology ws of key interest. Moreover, application of explicitly phylogenetic methods, such as phylogenetic PC analysis, will not change measures of disparity if the full variance in a dataset is considered [67], as was the case in this study.

## Competing interests

The authors declare that they have no competing interests.

## Authors’ contributions

CVB designed the analyses, conducted data collection and analyses, and drafted the manuscript; AG conceived of the study, designed the analyses, and drafted the manuscript. All authors read and approved the final manuscript.

## Supplementary Material

Additional file 1: Table S1 Principal component (PC) analysis scores for additional PCs. Click here for file

Additional file 2: Figure S2 Principal components (PC)1 to PC 4 for taxa grouped by diet as follows: (a,b) frugivores; (c,d) folivores; (e,f) omnivores; and (g,h) carnivores/insectivores. Symbols as in Figure 3 Click here for file

Additional file 3: Figure S3 Principal components (PC)1 to PC 4 for taxa grouped by habitat as follows: (a,b) arboreal; (c,d) terrestrial; and (e,f) fossorial. Symbols as in Figure 3. Click here for file

Additional file 4: Figure S4 Principal components (PC)1 to PC 4 for nocturnal taxa. Symbols as in Figure 3. Click here for file

Additional file 5: Table S5 List of taxa used in this study and ecological categories used in disparity analyses. *Used in the ‘maximum taxa’ dataset only. Click here for file
