# Supplementary figures and images for: Statistical support for the hypothesis of developmental constraint in marsupial skull evolution
Source: BMC Biol. 2013 Apr 26;11:52. doi: 10.1186/1741-7007-11-52 (PMC3660189; doi:10.1186/1741-7007-11-52)

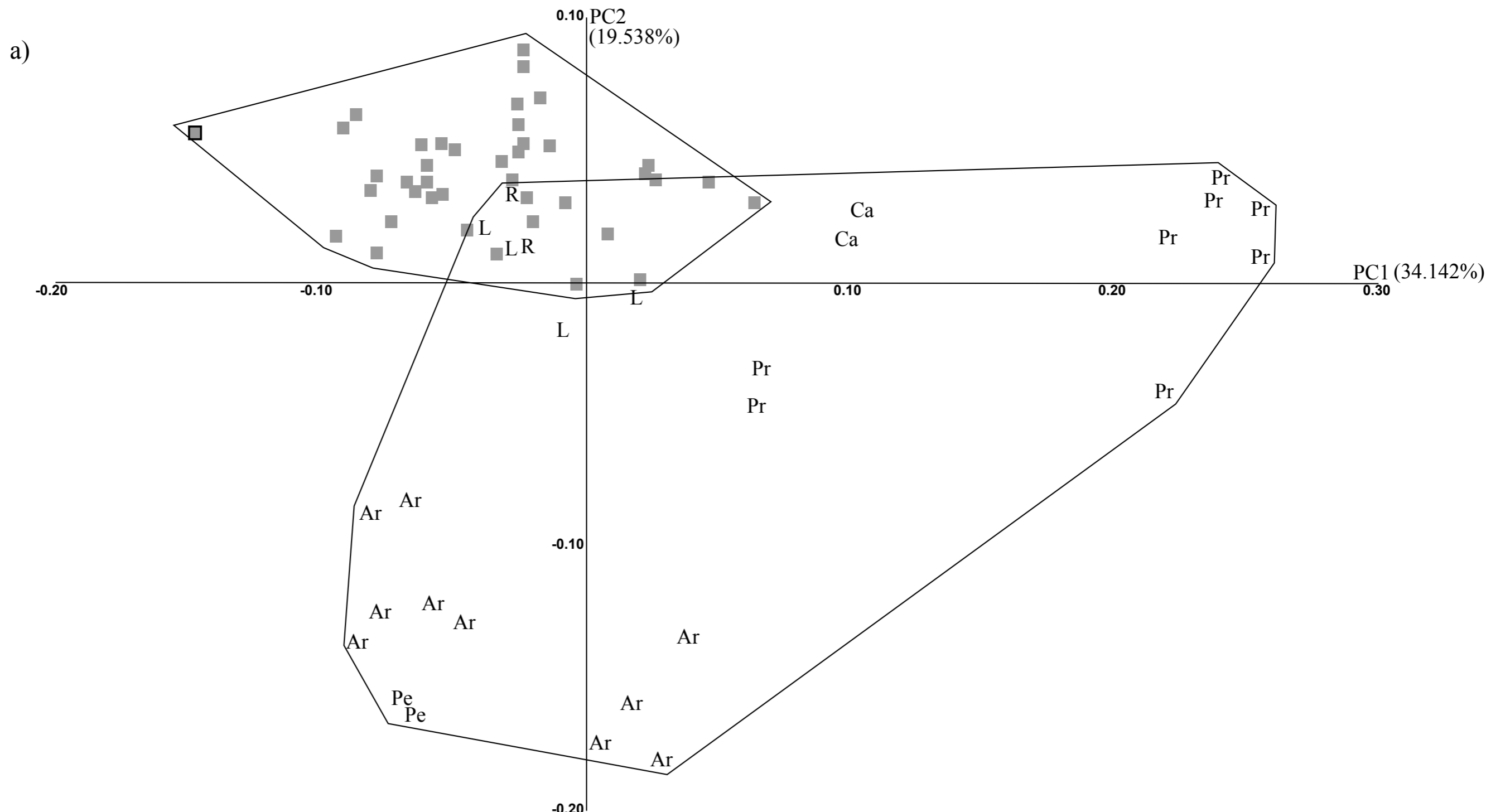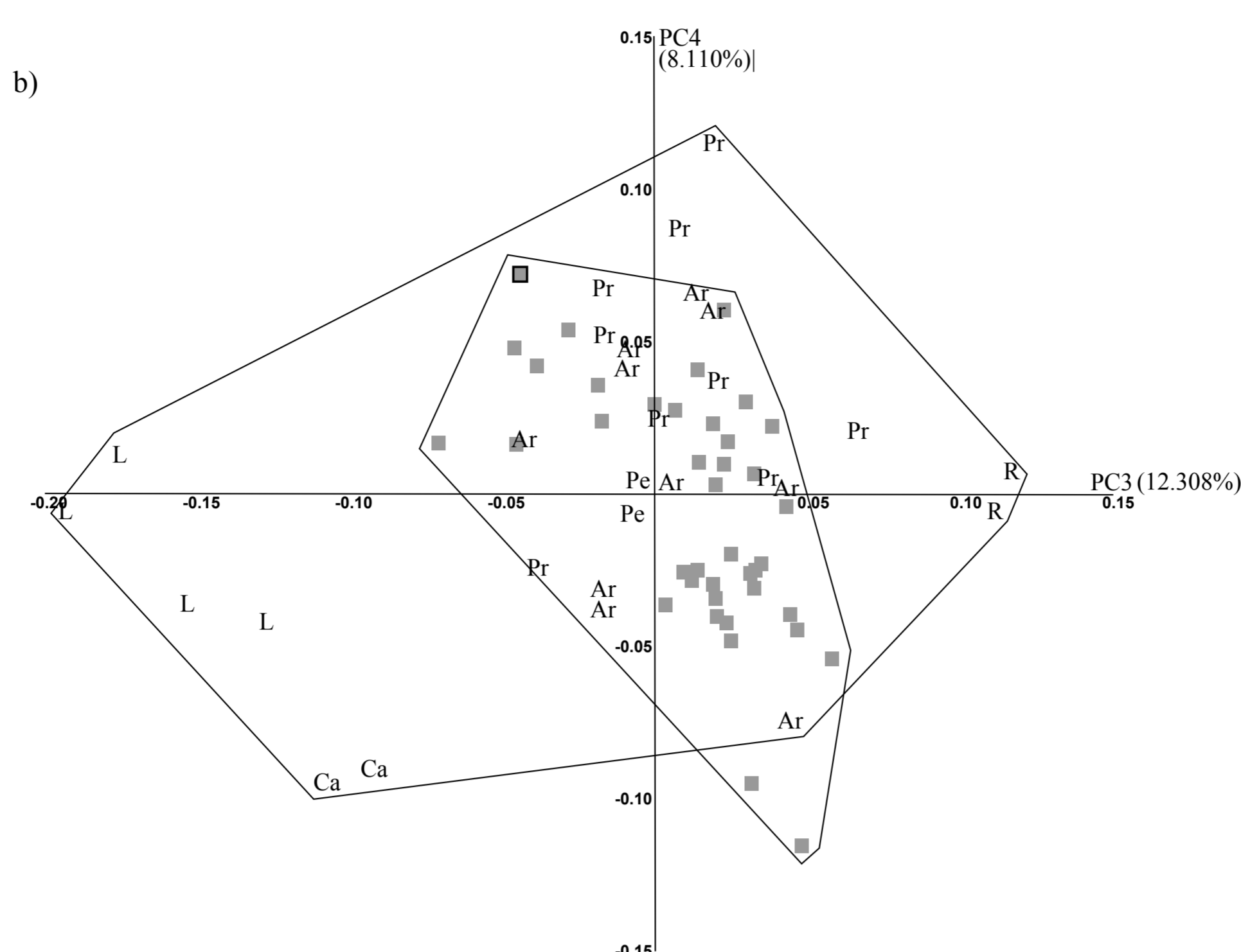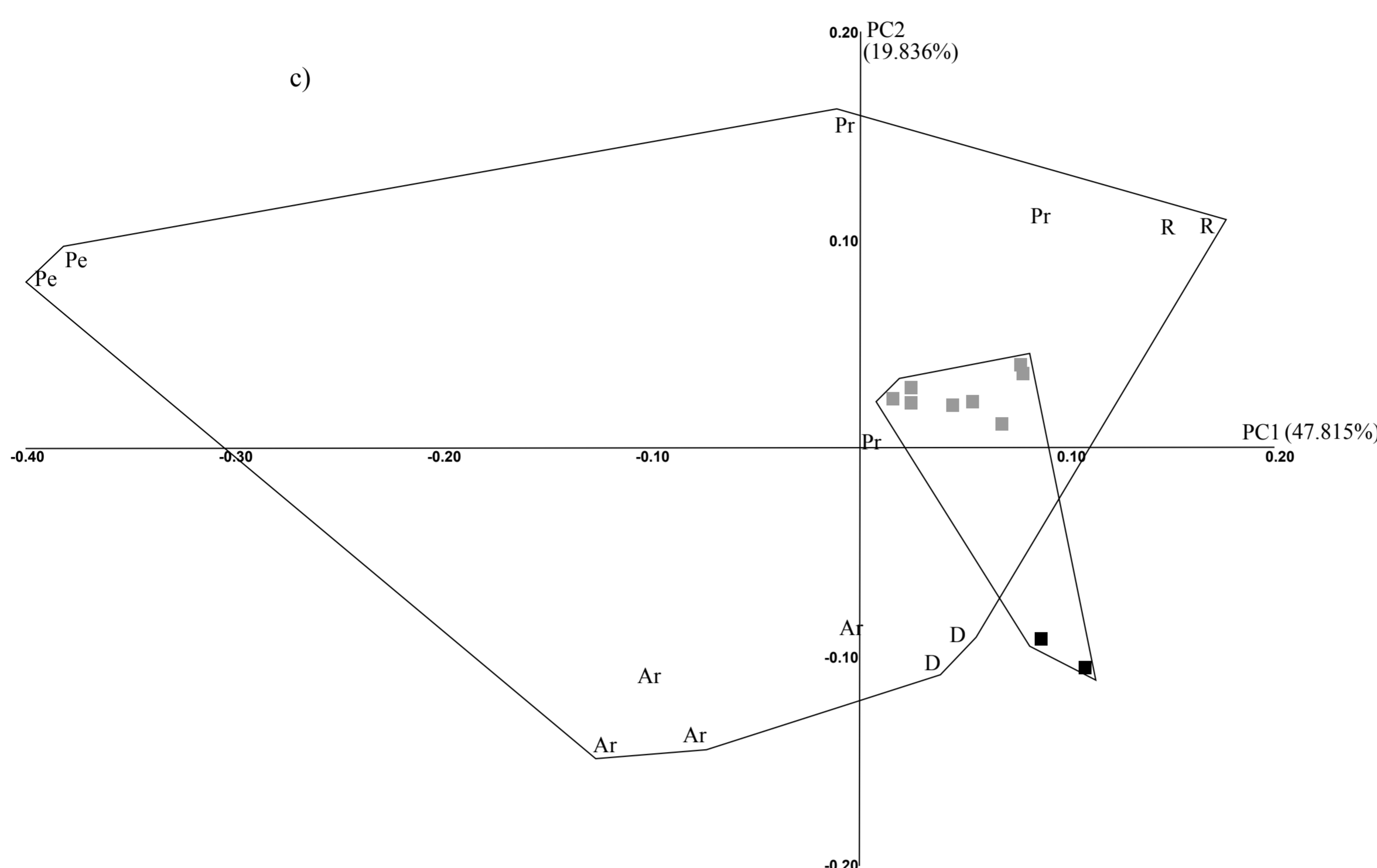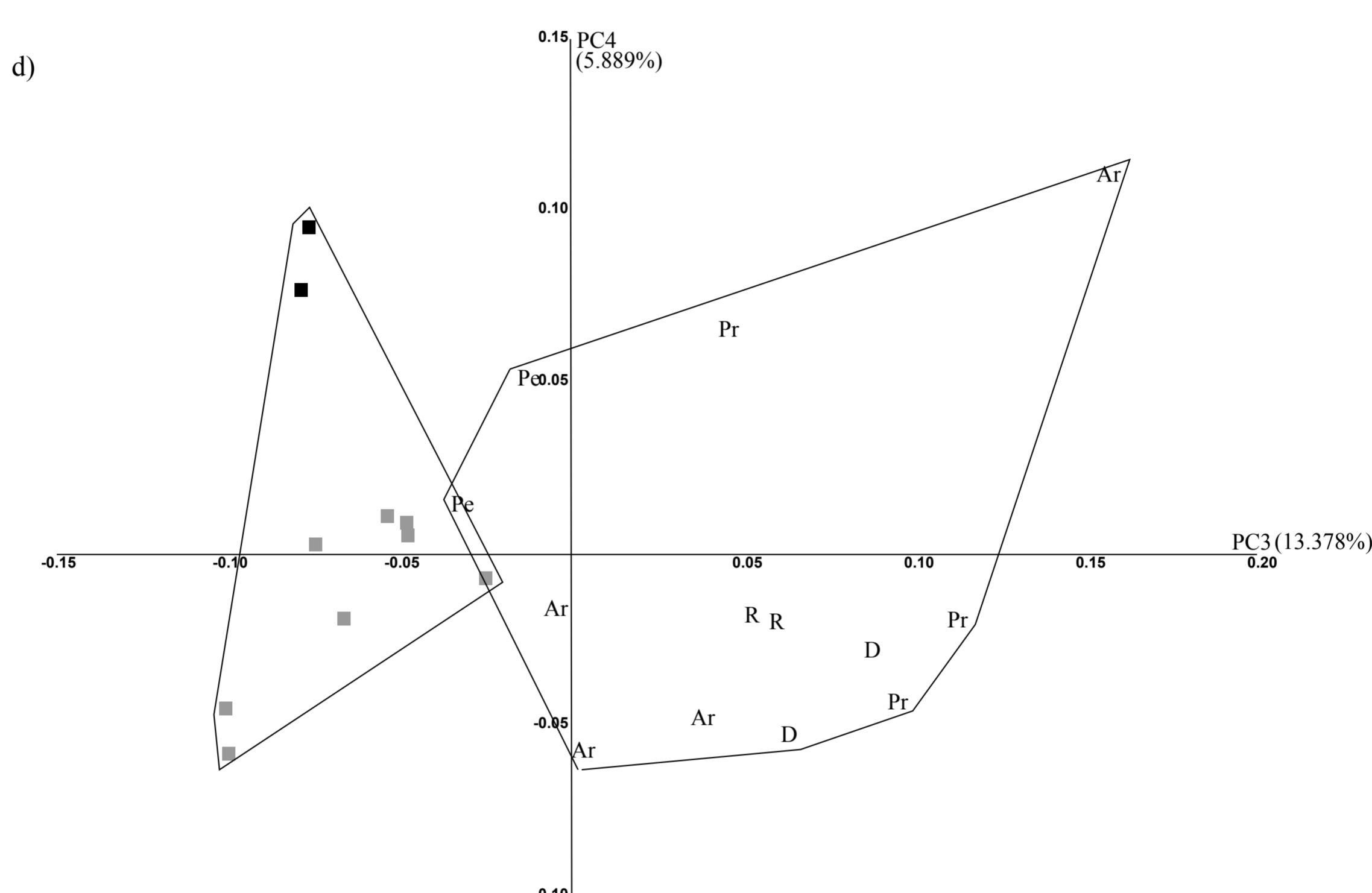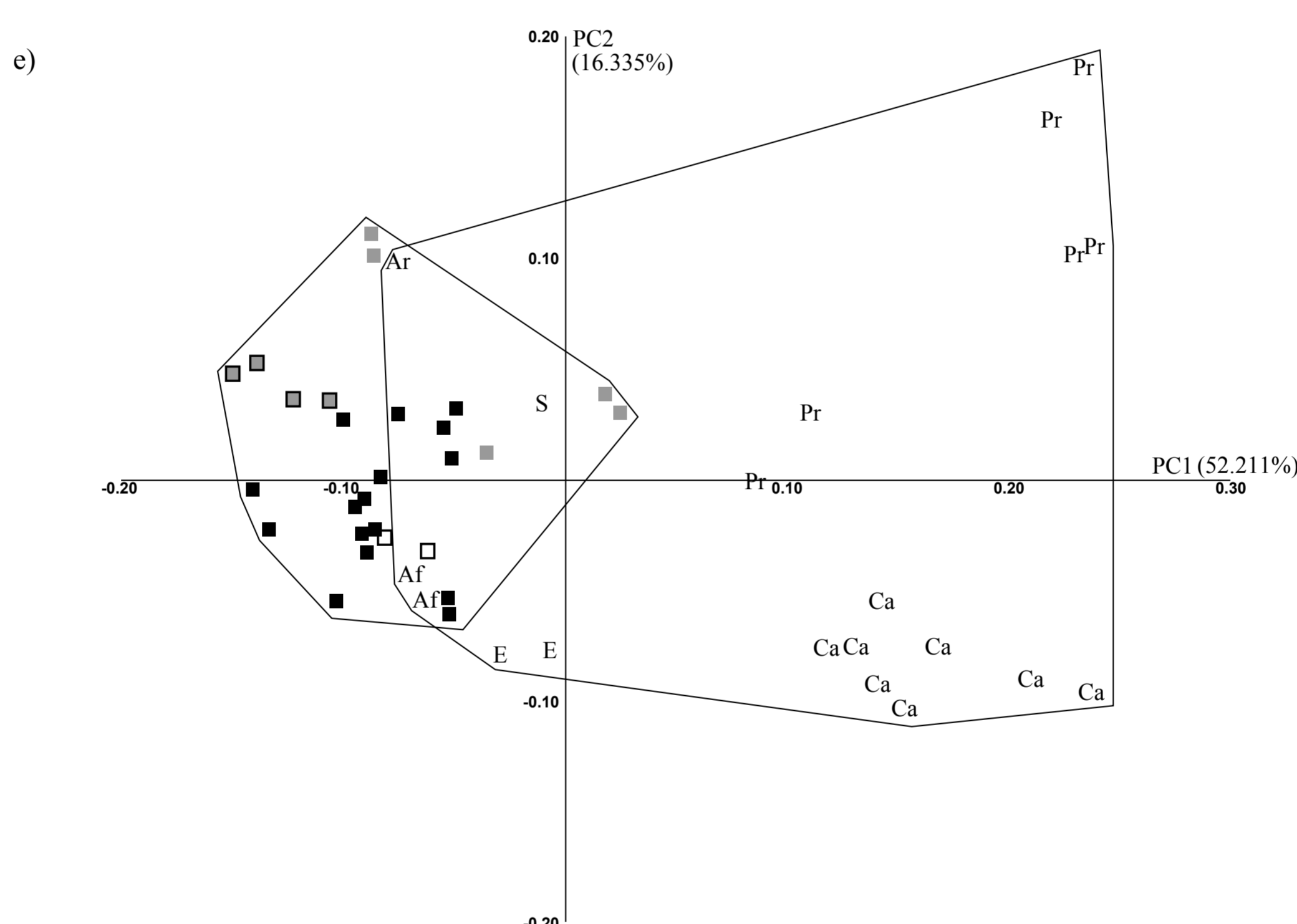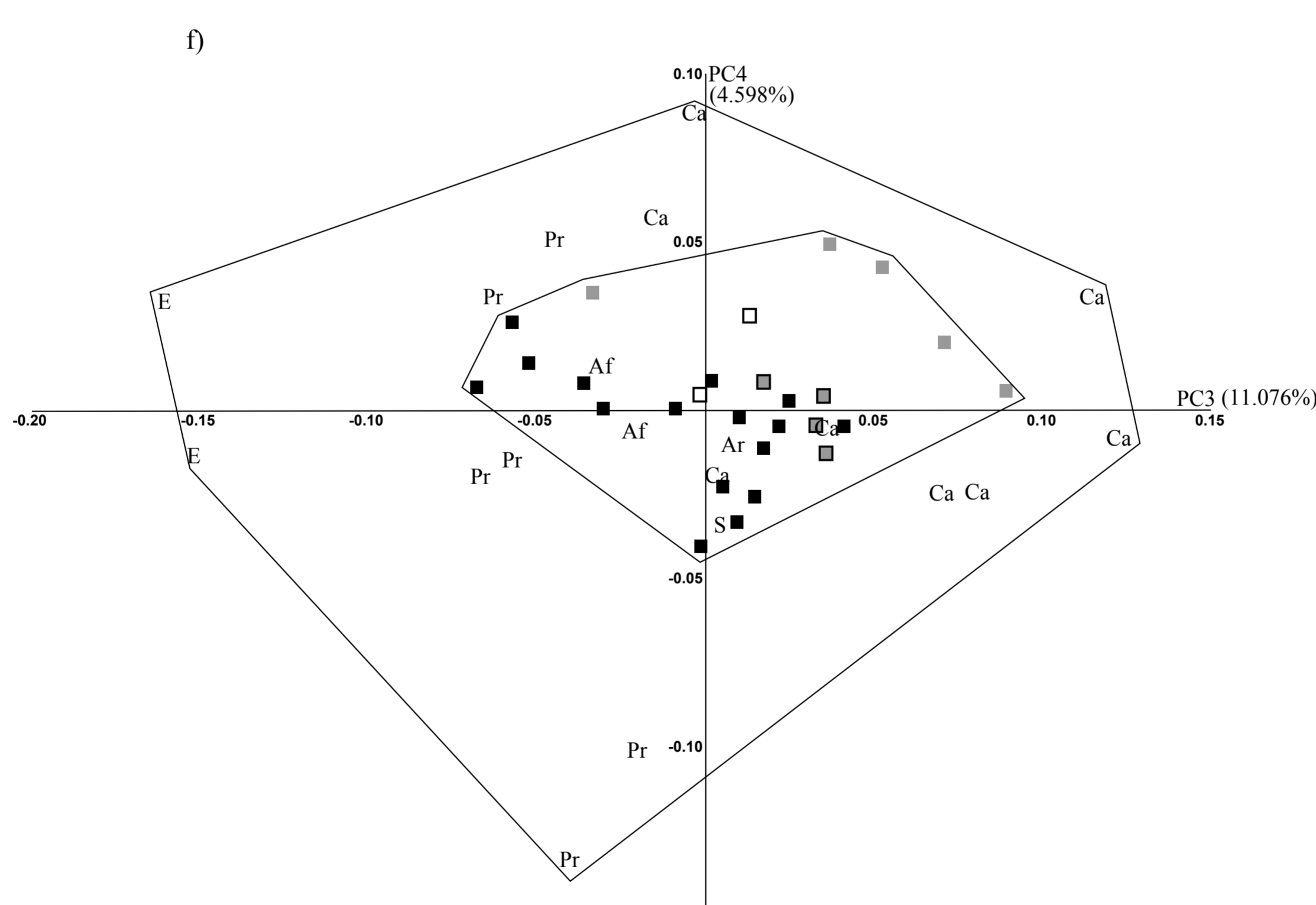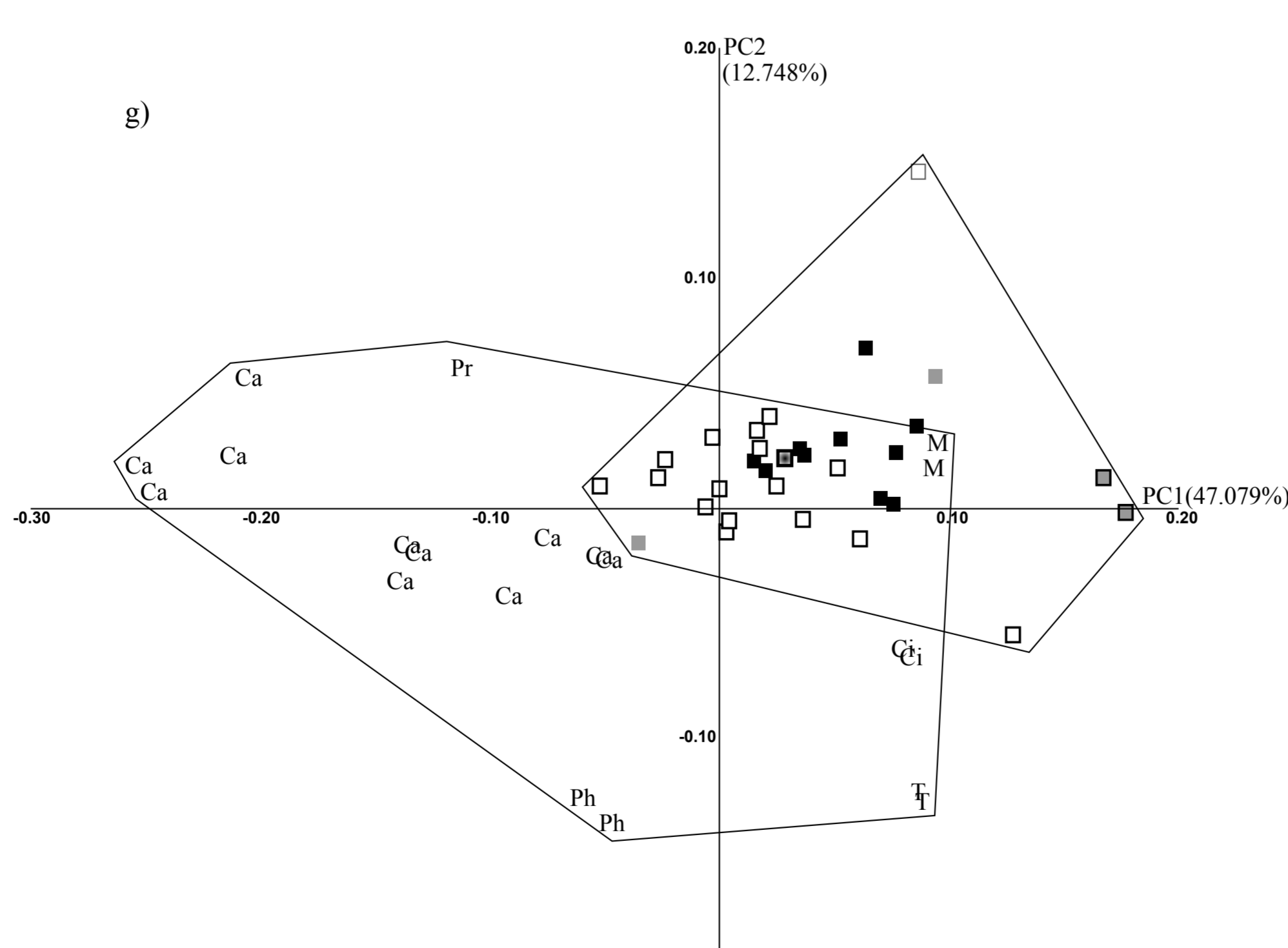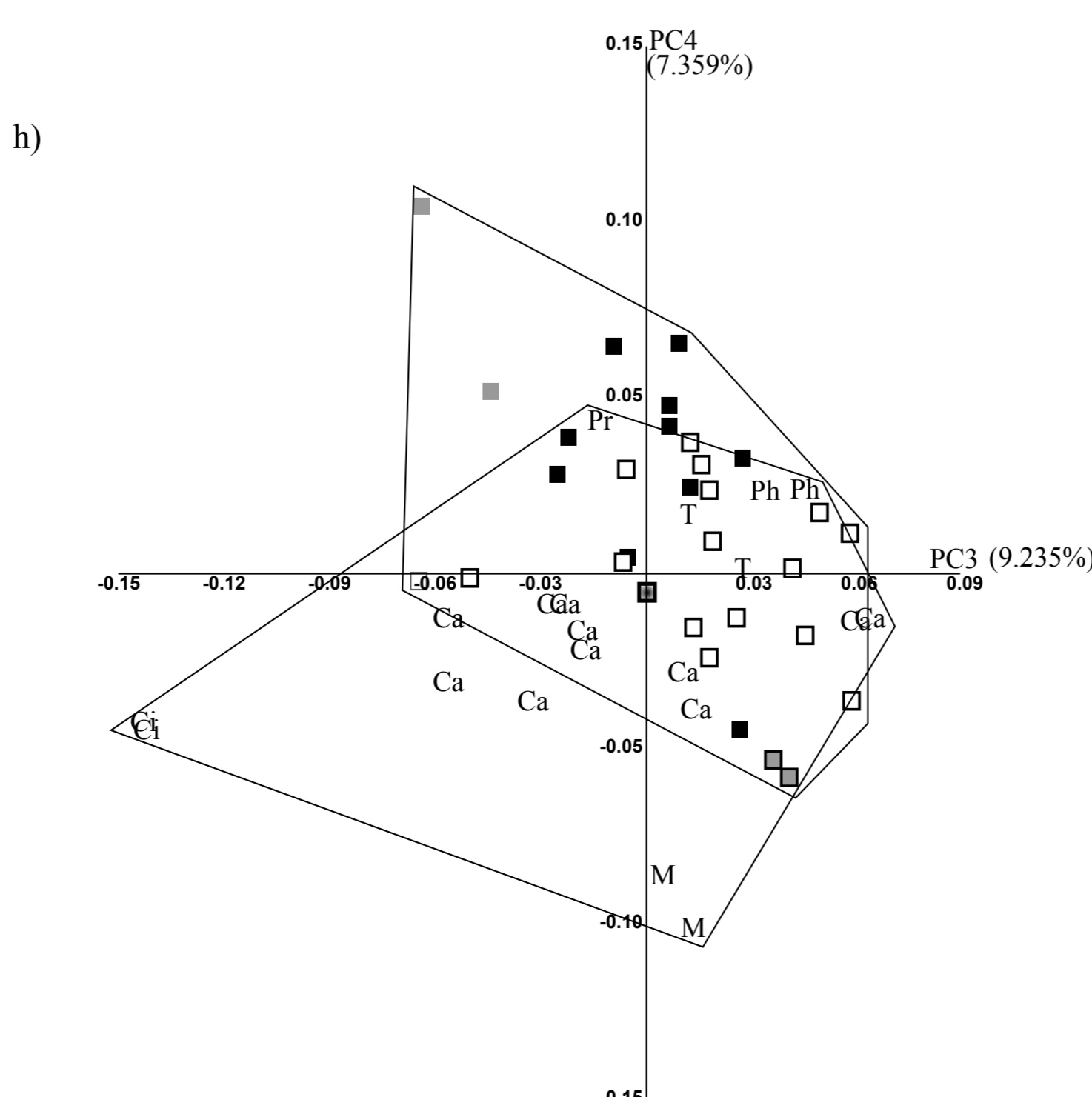

Supplement: Additional file 2: Figure S2 — Principal components (PC)1 to PC 4 for taxa grouped by diet as follows: (a,b) frugivores; (c,d) folivores; (e,f) omnivores; and (g,h) carnivores/insectivores. Symbols as in Figure 3 [file 1741-7007-11-52-S2.pdf]

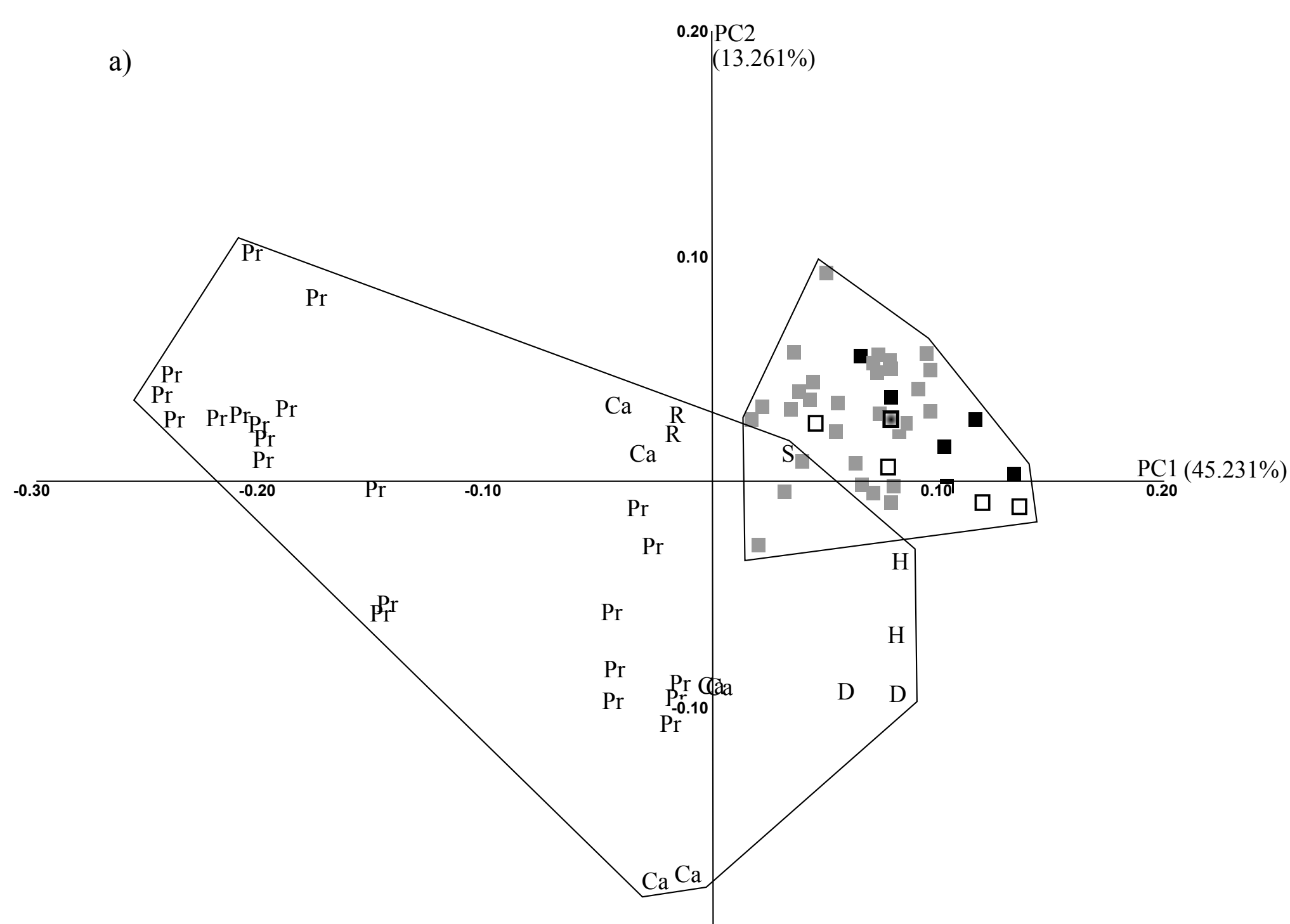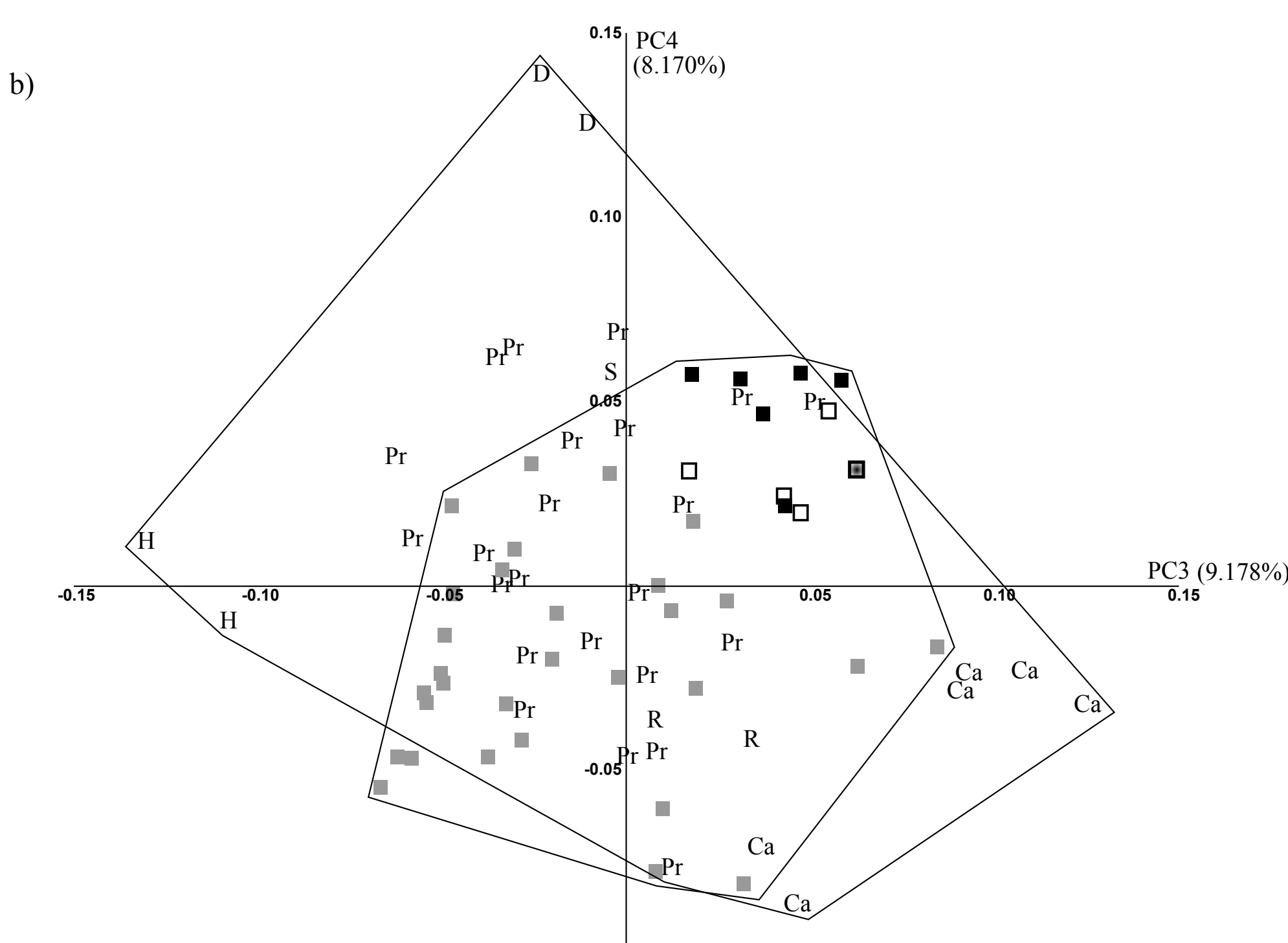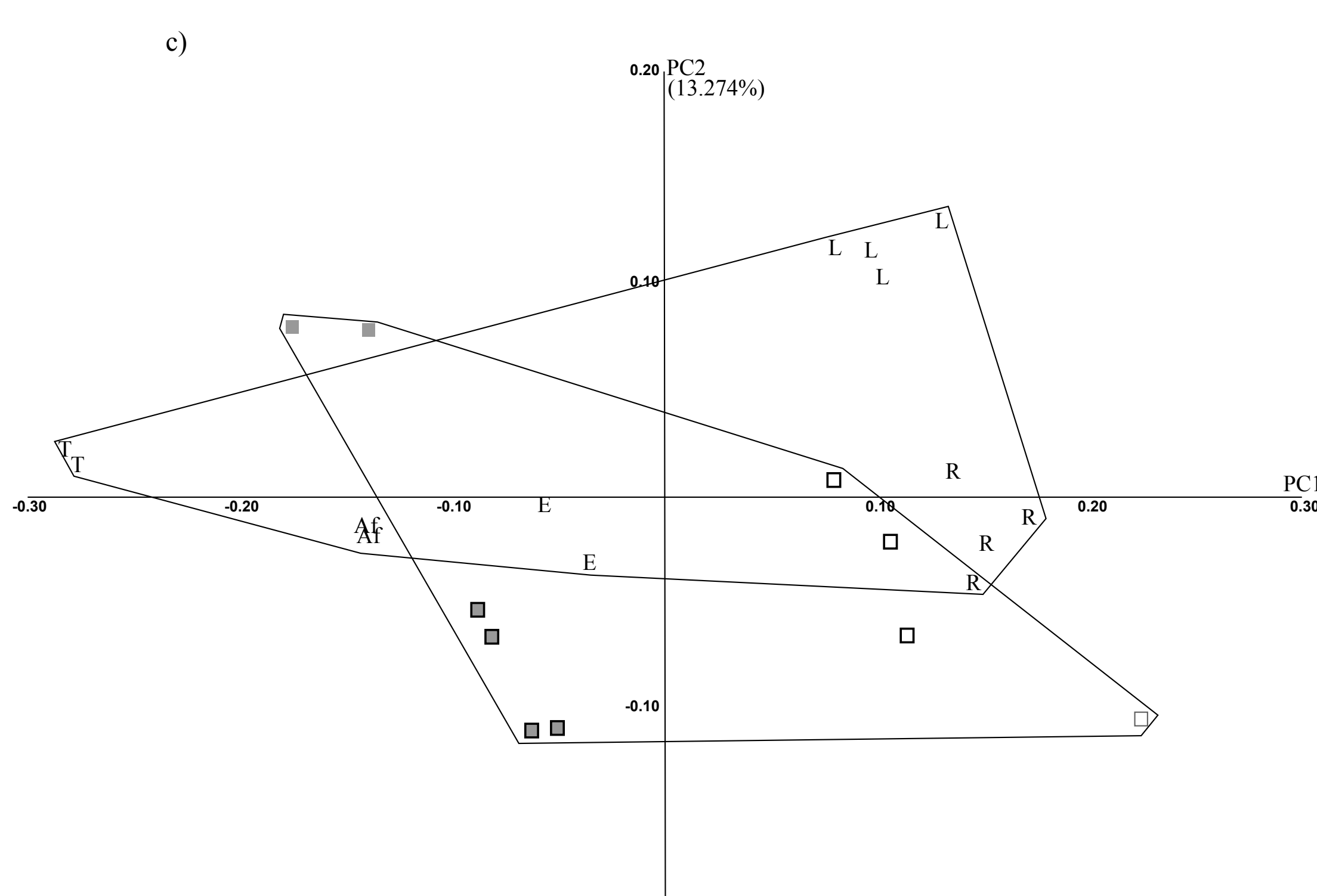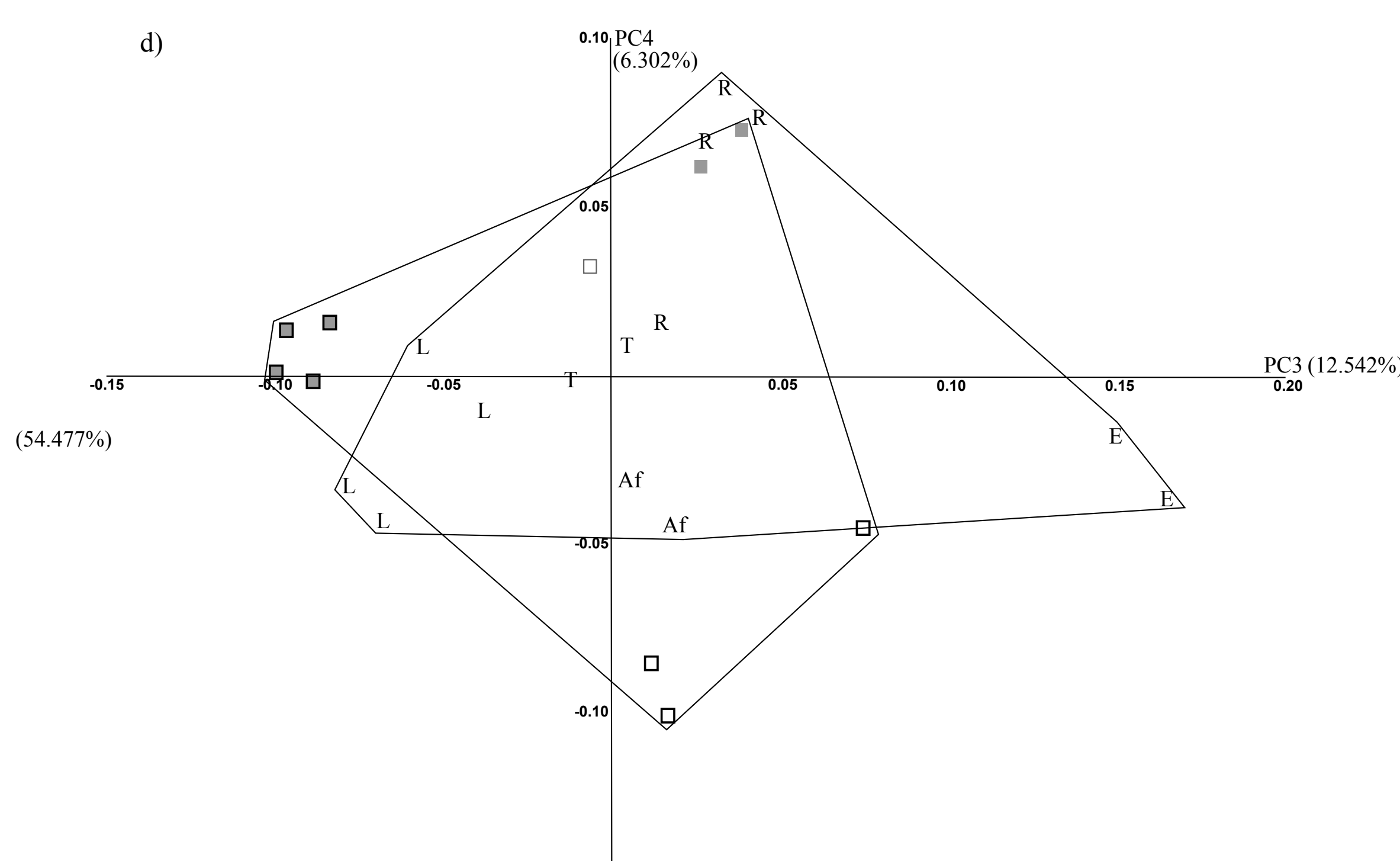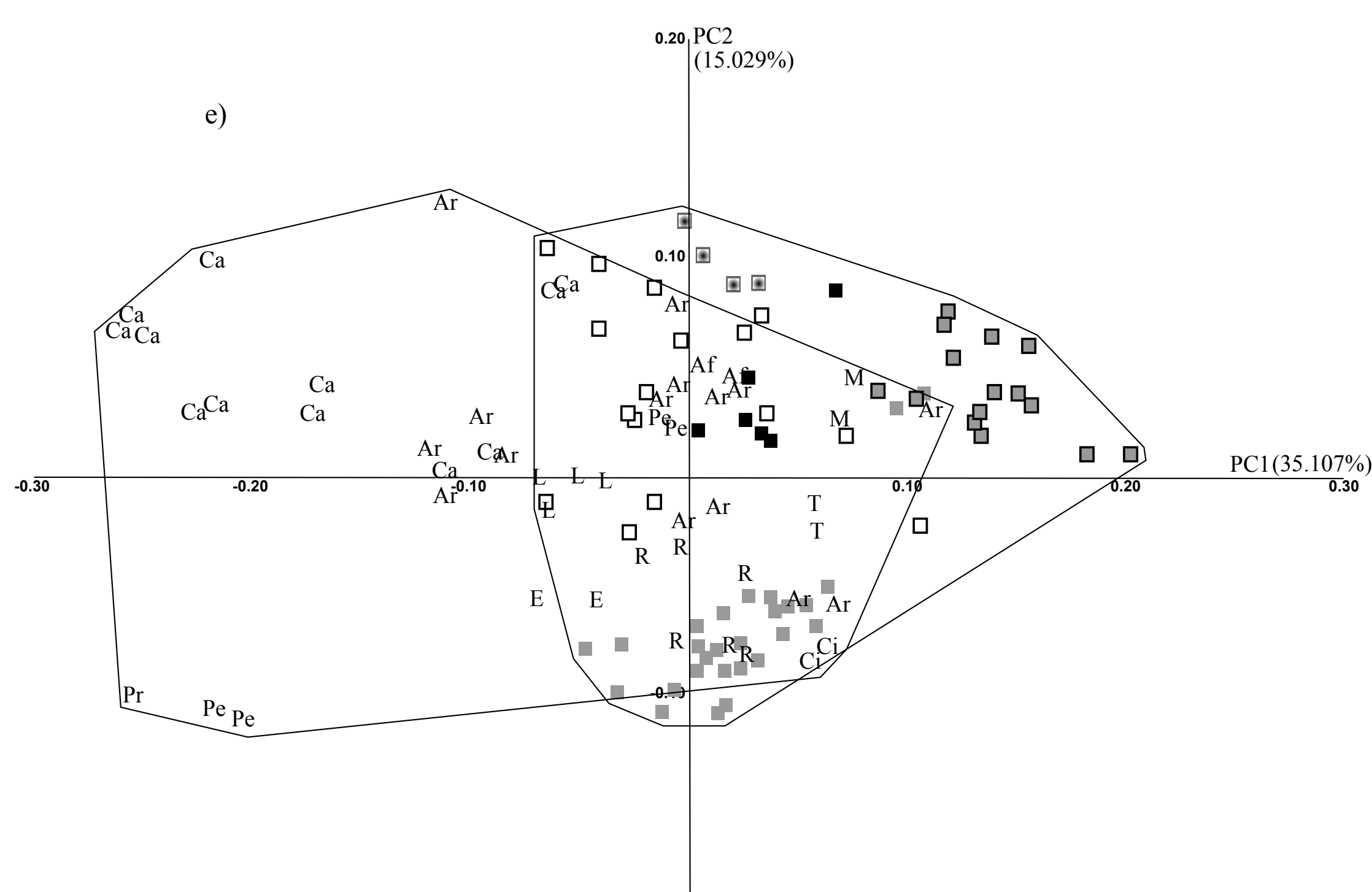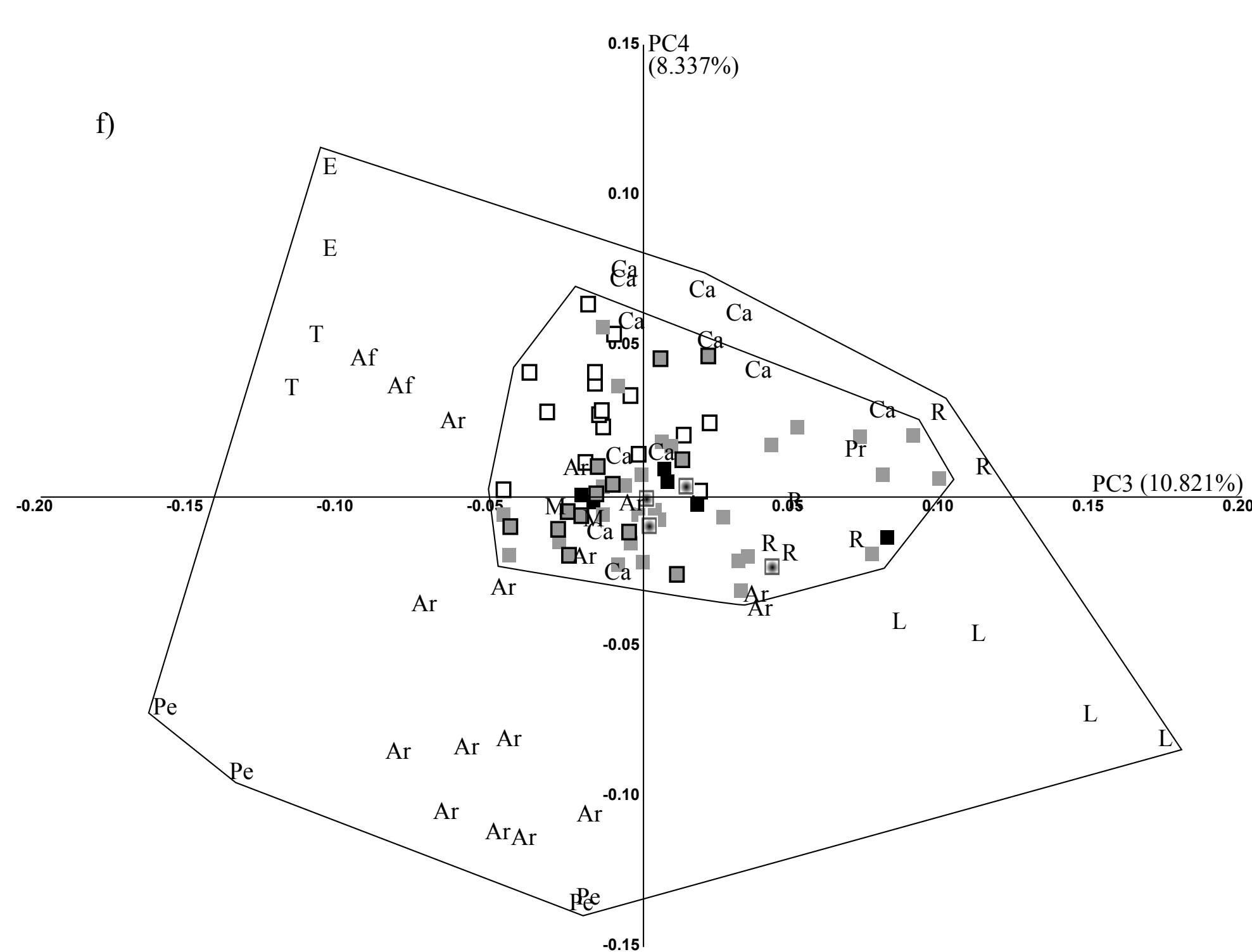

Supplement: Additional file 3: Figure S3 — Principal components (PC)1 to PC 4 for taxa grouped by habitat as follows: (a,b) arboreal; (c,d) terrestrial; and (e,f) fossorial. Symbols as in Figure 3. [file 1741-7007-11-52-S3.pdf]

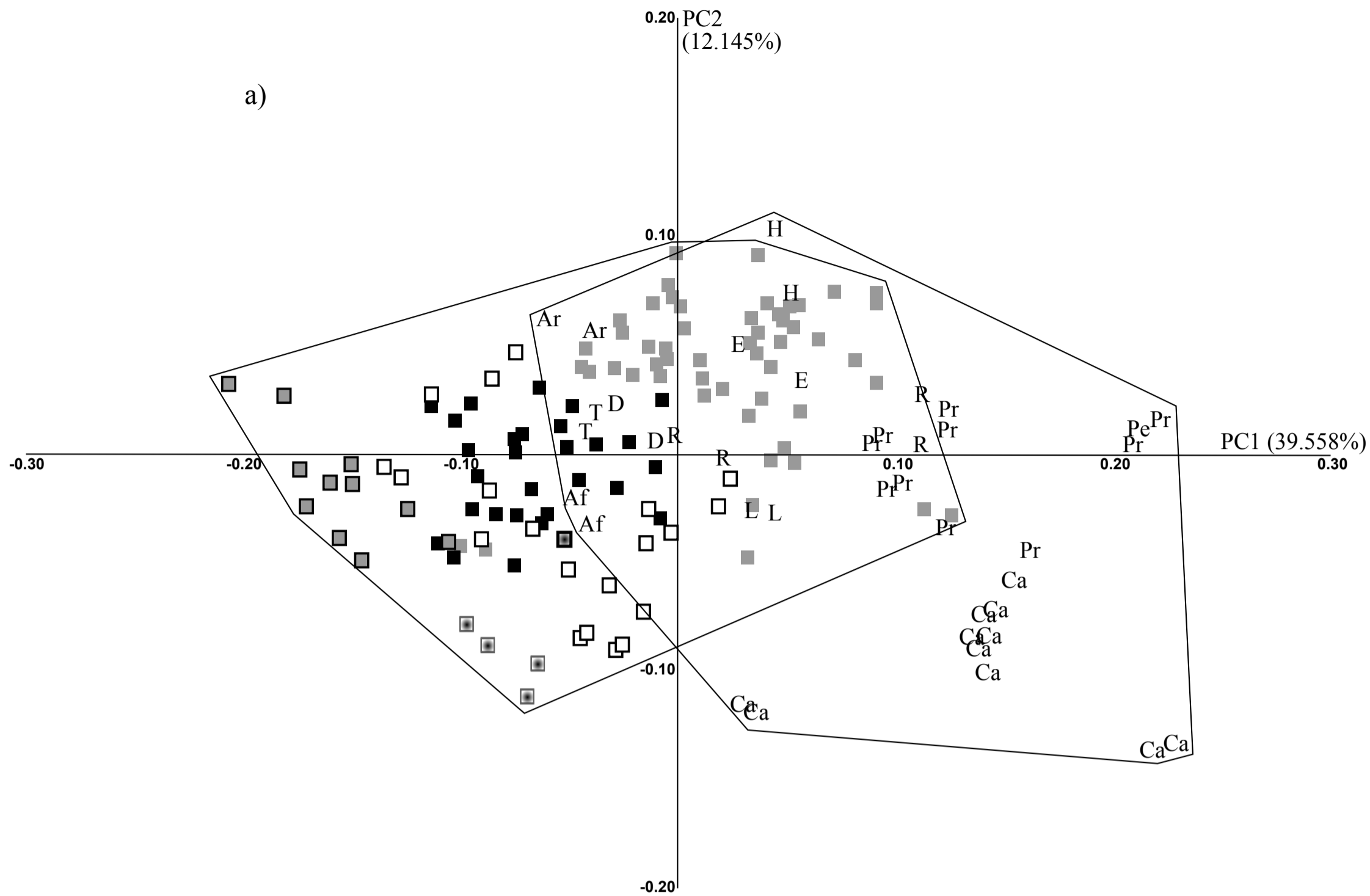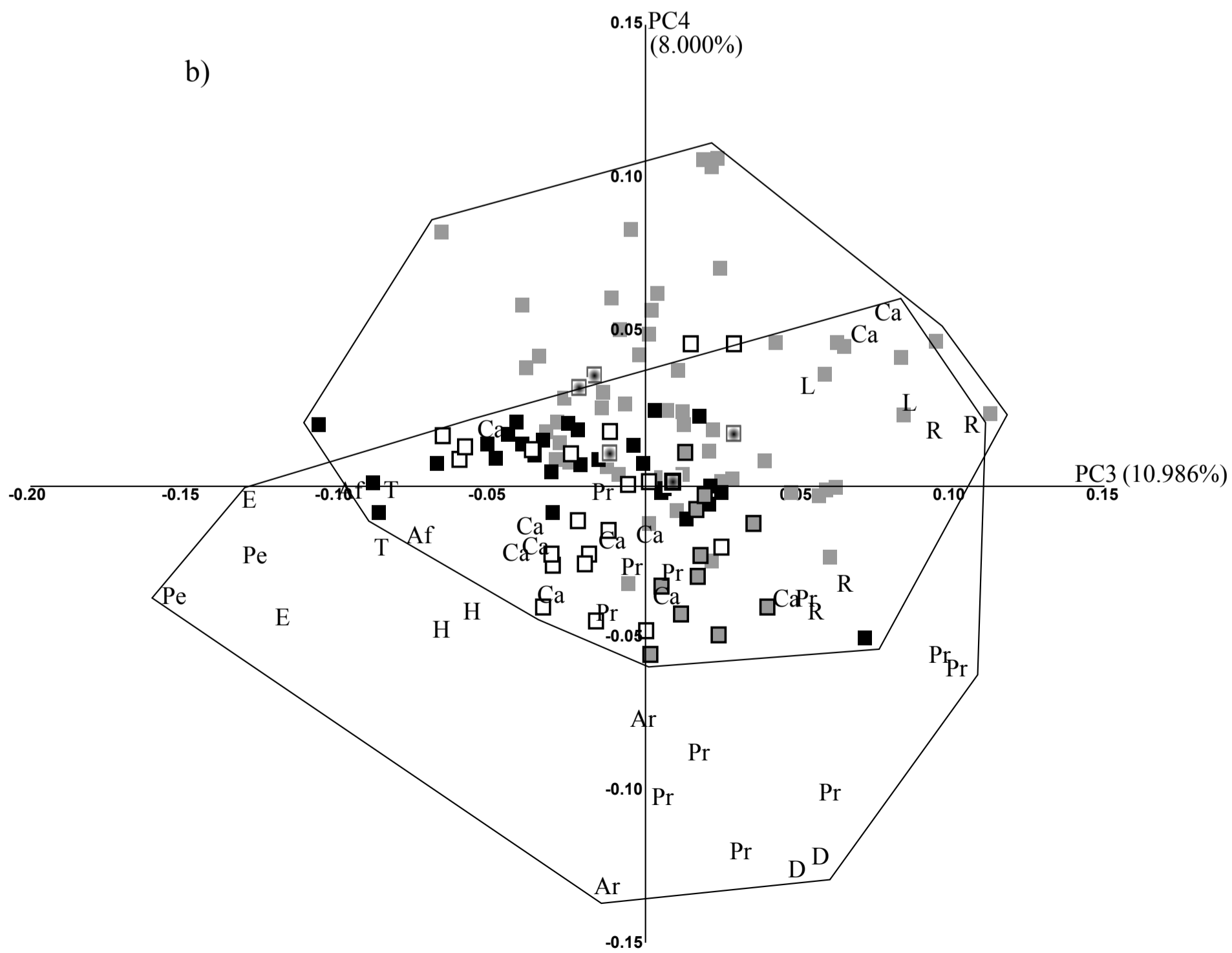

Supplement: Additional file 4: Figure S4 — Principal components (PC)1 to PC 4 for nocturnal taxa. Symbols as in Figure 3. [file 1741-7007-11-52-S4.pdf]
